# Supplementary material for: Reporting in clinical studies on platelet-rich plasma therapy among all medical specialties: A systematic review of Level I and II studies
Source: PLoS One. 2021 Apr 23;16(4):e0250007. doi: 10.1371/journal.pone.0250007 (PMC8064527; doi:10.1371/journal.pone.0250007)
Supplement: S1 Appendix — (DOCX) [file pone.0250007.s002.docx]

| Title | PMID | Level of Evidence | Journal Name | Field |
| --- | --- | --- | --- | --- |
| Injectable Platelet-, Leukocyte-, and Fibrin-Rich Plasma (iL-PRF) in the Management of Androgenetic Alopecia. | /pubmed/30045107 | 2 | Dermatologic Surgery | Cosmetic |
| Evaluation of carboxytherapy and platelet-rich plasma in treatment of periorbital hyperpigmentation: A comparative clinical trial. | /pubmed/29297970 | 2 | Journal of Cosmetic Dermatology | Cosmetic |
| Evaluation of autologous platelet-rich plasma plus ablative carbon dioxide fractional laser in the treatment of acne scars. | /pubmed/28853968 | 2 | Journal of cosmetic and laser therapy | Cosmetic |
| A randomized, double-blind, placebo- and active-controlled, half-head study to evaluate the effects of platelet-rich plasma on alopecia areata. | /pubmed/23607773 | 1 | British Journal of Dermatology | Cosmetic |
| Treatment of male pattern alopecia with platelet-rich plasma: A double-blind controlled study with analysis of platelet number and growth factor levels. | /pubmed/30287324 | 1 | Journal of the American Academy of Dermatology | Cosmetic |
| Autologous platelet rich plasma: topical versus intradermal after fractional ablative carbon dioxide laser treatment of atrophic acne scars. | /pubmed/24354616 | 2 | Dermatologic surgery | Cosmetic |
| The efficacy of autologous platelet rich plasma combined with ablative carbon dioxide fractional resurfacing for acne scars: a simultaneous split-face trial. | /pubmed/21635618 | 1 | Dermatologic Surgery | Cosmetic |
| Platelet-rich plasma versus CROSS technique with 100% trichloroacetic acid versus combined skin needling and platelet rich plasma in the treatment of atrophic acne scars: a comparative study. | /pubmed/25006854 | 2 | Dermatologic Surgery | Cosmetic |
| Efficacy of autologous platelet-rich plasma combined with fractional ablative carbon dioxide resurfacing laser in treatment of facial atrophic acne scars: A split-face randomized clinical trial. | pubmed/26924405 | 1 | Indian Journal of Dermatology, Venereology, and Leprosy | Cosmetic |
| Double-blind clinical trial to compare autologous fat grafts versus autologous fat grafts with PDGF: no effect of PDGF. | /pubmed/25068344 | 2 | Plastic and Reconstructive Surgery | Cosmetic |
| The Effect of Platelet-Rich Plasma in Hair Regrowth: A Randomized Placebo-Controlled Trial. | /pubmed/26400925 | 1 | Stem Cells Translational Medicine | Cosmetic |
| Efficacy of platelet-rich plasma in the treatment of androgenetic (male-patterned) alopecia: A pilot randomized controlled trial. | /pubmed/27593381 | 1 | Journal of Cosmetic and Laser Therapy | Cosmetic |
| Clinical and immunohistochemical comparative study of the efficacy of carboxytherapy vs platelet-rich plasma in treatment of stretch marks. | /pubmed/29316152 | 2 | Journal of Cosmetic Dermatology | Cosmetic |
| Therapeutic effect of micro needling and autologous platelet-rich plasma in the treatment of atrophic scars: A randomized study. | /pubmed/28504480 | 2 | Journal of Cosmetic Dramatology | Cosmetic |
| The effect of autologous activated platelet-rich plasma injection on female pattern hair loss: A randomized placebo-controlled study. | /pubmed/28503741 | 1 | Journal of Cosmetic Dramatology | Cosmetic |
| Double-Blind, Placebo-Controlled Pilot Study on the Use of Platelet-Rich Plasma in Women with Female Androgenetic Alopecia. | /pubmed/27608205 | 1 | Dermatologic Surgery | Cosmetic |
| Platelets rich plasma versus minoxidil 5% in treatment of alopecia areata: A trichoscopic evaluation. | /pubmed/27791311 | 1 | Dermatologic Therapy | Cosmetic |
| Comparison between the efficacy and safety of platelet-rich plasma vs. microdermabrasion in the treatment of striae distensae: clinical and histopathological study. | /pubmed/26147455 | 1 | Journal of Cosmetic Dermatology | Cosmetic |
| Platelet-Rich Plasma Versus Tretinoin in Treatment of Striae Distensae: A Comparative Study. | /pubmed/29701622 | 2 | Dermatologic Surgery | Cosmetic |
| The effect of platelet-rich plasma on the outcome of short-term narrowband-ultraviolet B phototherapy in the treatment of vitiligo: a pilot study. | /pubmed/26695436 | 2 | Journal of Cosmetic Dermatology | Dermatology |
| Clinical efficacy of platelet rich plasma in combination with methotrexate in chronic plaque psoriatic patients. | /pubmed/27418241 | 2 | Dermatologic Therapy | Dermatology |
| Combined treatment with fractional carbon dioxide laser, autologous platelet-rich plasma, and narrow band ultraviolet B for vitiligo in different body sites: A prospective, randomized comparative trial. | /pubmed/28834191 | 1 | Journal of Cosmetic Dermatology | Dermatology |
| Comparing hybrid hyaluronic acid with PRP in end career athletes with degenerative cartilage lesions of the knee. | /pubmed/28002896 | 1 | Journal of Biological Regulators and Homeostatic Agents | Musculoskeletal |
| Leukocyte-poor platelet-rich plasma is more effective than the conventional therapy with acetaminophen for the treatment of early knee osteoarthritis. | /pubmed/27506585 | 1 | Archives of Orthopaedic and Trauma Surgery | Musculoskeletal |
| Platelet rich plasma versus corticosteroid injection for plantar fasciitis: A comparative study. | /pubmed/26362235 | 1 | The Foot | Musculoskeletal |
| Platelet-Rich Plasma Has Better Long-Term Results Than Corticosteroids or Placebo for Chronic Plantar Fasciitis: Randomized Control Trial. | /pubmed/30448183 | 2 | The Journal of Foot and Ankle surgery | Musculoskeletal |
| Platelet-rich plasma efficacy versus corticosteroid injection treatment for chronic severe plantar fasciitis. | /pubmed/24419823 | 1 | Foot and Ankle International | Musculoskeletal |
| Steroid vs. Platelet-Rich Plasma in Ultrasound-Guided Sacroiliac Joint Injection for Chronic Low Back Pain. | /pubmed/27677100 | 1 | Pain Practice | Musculoskeletal |
| Plantar Fasciitis-A Comparison of Treatment with Intralesional Steroids versus Platelet-Rich Plasma (A Randomized, Blinded Study). | /pubmed/27726423 | 1 | Journal of the American Podiatric Medical Association | Musculoskeletal |
| Chronic Plantar Fasciitis: Effect of Platelet-Rich Plasma, Corticosteroid, and Placebo. | /pubmed/26913766 | 1 | Orthopeadics | Musculoskeletal |
| Ongoing positive effect of platelet-rich plasma versus corticosteroid injection in lateral epicondylitis: a double-blind randomized controlled trial with 2-year follow-up. | /pubmed/21422467 | 1 | The American Journal of Sports Medicine | Musculoskeletal |
| A Prospective Study Comparing Platelet-Rich Plasma and Local Anesthetic (LA)/Corticosteroid in Intra-Articular Injection for the Treatment of Lumbar Facet Joint Syndrome. | /pubmed/27989008 | 1 | Pain Practice | Musculoskeletal |
| The effects of injecting intra-articular platelet-rich plasma or prolotherapy on pain score and function in knee osteoarthritis. | /pubmed/29379278 | 2 | Clinical interventions in Aging | Musculoskeletal |
| Autologous platelet-rich plasma versus dextrose prolotherapy for the treatment of chronic recalcitrant plantar fasciitis. | /pubmed/23876935 | 1 | PM&R | Musculoskeletal |
| Effectiveness of Four Different Treatment Modalities in the Treatment of Chronic Plantar Fasciitis During a 36-Month Follow-Up Period: A Randomized Controlled Trial. | /pubmed/30149850 | 1 | The Journal of Foot and Ankle surgery | Musculoskeletal |
| Platelet-rich plasma or hyaluronate in the management of osteochondral lesions of the talus. | /pubmed/22253252 | 2 | The American Journal of Sports Medicine | Musculoskeletal |
| Ultrasound-Guided Injection of Platelet-Rich Plasma and Hyaluronic Acid, Separately and in Combination, for Hip Osteoarthritis: A Randomized Controlled Study. | /pubmed/26797697 | 1 | The American Journal of Sports medicine | Musculoskeletal |
| Platelet-rich plasma intra-articular injection versus hyaluronic acid viscosupplementation as treatments for cartilage pathology: from early degeneration to osteoarthritis. | /pubmed/21831567 | 2 | Arthroscopy: The Journal of Arthroscopic & Related Surgery | Musculoskeletal |
| Comparison of hyaluronic acid and PRP intra-articular injection with combined intra-articular and intraosseous PRP injections to treat patients with knee osteoarthritis. | /pubmed/29388085 | 2 | Clinical Rheumatology | Musculoskeletal |
| Efficacy of ultrasound-guided intra-articular injections of platelet-rich plasma versus hyaluronic acid for hip osteoarthritis. | /pubmed/24579221 | 1 | Orthopedics | Musculoskeletal |
| Comparison between hyaluronic acid and platelet-rich plasma, intra-articular infiltration in the treatment of gonarthrosis. | /pubmed/23104611 | 1 | The American Journal of Sports Medicine | Musculoskeletal |
| Choice of intra-articular injection in treatment of knee osteoarthritis: platelet-rich plasma, hyaluronic acid or ozone options. | /pubmed/27056686 | 1 | Knee Surgery, Sports Traumatology, Arthroscopy | Musculoskeletal |
| Treatment of knee joint osteoarthritis with autologous platelet-rich plasma in comparison with hyaluronic acid. | /pubmed/22513879 | 2 | American Journal of Physical Medicine & Rehabilitation | Musculoskeletal |
| Intra-articular hyaluronic acid vs platelet-rich plasma in the treatment of hip osteoarthritis. | /pubmed/27981279 | 1 | Medical Ultrasonography | Musculoskeletal |
| Intra-Articular Injections of Platelet-Rich Plasma versus Hyaluronic Acid in the Treatment of Osteoarthritic Knee Pain: A Randomized Clinical Trial in the Context of the Spanish National Health Care System. | /pubmed/27384560 | 1 | International Journal of Molecular Sciences | Musculoskeletal |
| Clinical results of hip arthroscopy for labral tears: a comparison between intraoperative platelet-rich plasma and bupivacaine injection. | /pubmed/25442663 | 2 | Arthroscopy: The Journal of Arthroscopic & Related Surgery | Musculoskeletal |
| A randomized study of autologous conditioned plasma and steroid injections in the treatment of lateral epicondylitis. | /pubmed/26224613 | 1 | International Orthopeadics | Musculoskeletal |
| Intra-articular injections of expanded mesenchymal stem cells with and without addition of platelet-rich plasma are safe and effective for knee osteoarthritis. | /pubmed/29511819 | 2 | knee Surgery, Sports Traumatology, Arthroscopy | Musculoskeletal |
| Autologous proliferative therapies in recalcitrant lateral epicondylitis. | /pubmed/25357145 | 2 | American Journal of Physical Medicine & Rehabilitation. | Musculoskeletal |
| Lumbar Intradiskal Platelet-Rich Plasma (PRP) Injections: A Prospective, Double-Blind, Randomized Controlled Study. | /pubmed/26314234 | 1 | PM&R | Musculoskeletal |
| Does intraoperative application of leukocyte-poor platelet-rich plasma during arthroscopy for knee degeneration affect postoperative pain, function and quality of life? A 12-month randomized controlled double-blind trial. | /pubmed/25957981 | 2 | Archives of Orthopaedic and Trauma Surgery | Musculoskeletal |
| Intra-articular Autologous Conditioned Plasma Injections Provide Safe and Efficacious Treatment for Knee Osteoarthritis: An FDA-Sanctioned, Randomized, Double-blind, Placebo-controlled Clinical Trial. | /pubmed/26831629 | 1 | The American Journal of Sports medicine | Musculoskeletal |
| Effects of Platelet-Rich Plasma on Pain and Muscle Strength in Patients with Knee Osteoarthritis. | /pubmed/29210705 | 1 | American Journal of Physical Medicine & Rehabilitation | Musculoskeletal |
| Autologous Conditioned Plasma Versus Placebo Injection Therapy in Lateral Epicondylitis of the Elbow: A Double Blind, Randomized Study. | /pubmed/28222465 | 2 | Sportverletz Sportschaden | Musculoskeletal |
| Multiple PRP injections are more effective than single injections and hyaluronic acid in knees with early osteoarthritis: a randomized, double-blind, placebo-controlled trial. | /pubmed/26233594 | 1 | Knee Surgery, Sports Traumatology, Arthroscopy | Musculoskeletal |
| Treatment with platelet-rich plasma is more effective than placebo for knee osteoarthritis: a prospective, double-blind, randomized trial. | /pubmed/23299850 | 1 | American Journal of Sports Medicine | Musculoskeletal |
| Inefficacy of ultrasound-guided local injections of autologous conditioned plasma for recent epicondylitis: results of a double-blind placebo-controlled randomized clinical trial with one-year follow-up. | /pubmed/26350485 | 1 | Rheumatology | Musculoskeletal |
| Clinical Effects of Platelet-Rich Plasma and Hyaluronic Acid as an Additional Therapy for Talar Osteochondral Lesions Treated with Microfracture Surgery: A Prospective Randomized Clinical Trial. | /pubmed/25825393 | 1 | Foot and Ankle International | Musculoskeletal |
| Platelet-rich plasma versus autologous whole blood for the treatment of chronic lateral elbow epicondylitis: a randomized controlled clinical trial. | /pubmed/21813443 | 1 | The American Journal of Sports Medicine | Musculoskeletal |
| Use of platelet-rich plasma for the treatment of refractory jumper's knee. | /pubmed/19641918 | 2 | International Orthopaedics | Musculoskeletal |
| Platelet-rich plasma versus focused shock waves in the treatment of jumper's knee in athletes. | /pubmed/23408591 | 1 | American Journal of Sports Medicine | Musculoskeletal |
| Treatment of chronic elbow tendinosis with buffered platelet-rich plasma. | /pubmed/16735582 | 2 | The American Journal of Sports Medicine | Musculoskeletal |
| Platelet-rich plasma as a treatment for patellar tendinopathy: a double-blind, randomized controlled trial. | /pubmed/24481828 | 1 | The American Journal of Sports Medicine | Musculoskeletal |
| Platelet-rich plasma injection for chronic Achilles tendinopathy: a randomized controlled trial. | /pubmed/20068208 | 1 | The Journal of the American Medical Association | Musculoskeletal |
| Growth factor-based therapies provide additional benefit beyond physical therapy in resistant elbow tendinopathy: a prospective, single-blind, randomized trial of autologous blood injections versus platelet-rich plasma injections. | /pubmed/21406450 | 1 | British Journal of Sports medicine | Musculoskeletal |
| Ultrasound-Guided Intratendinous Injections with Platelet-Rich Plasma or Autologous Whole Blood for Treatment of Proximal Hamstring Tendinopathy: A Double-Blind Randomized Controlled Trial. | /pubmed/26206832 | 1 | Journal of Ultrasound in Medicine | Musculoskeletal |
| The comparison of the effect of corticosteroids and platelet-rich plasma (PRP) for the treatment of plantar fasciitis. | /pubmed/22399039 | 2 | Archives of Orthopaedic and Trauma Surgery | Musculoskeletal |
| Platelet-rich plasma injection is more effective than hyaluronic acid in the treatment of knee osteoarthritis. | /pubmed/24119476 | 2 | ACTA CHIRURGIAE ORTHOPAEDICAEET TRAUMATOLOGIAE ČECHOSL | Musculoskeletal |
| Role of isolated percutaneous autologous platelet concentrate in delayed union of long bones. | /pubmed/29167980 | 1 | European Journal of Orthopaedic Surgery & Traumatology | Musculoskeletal |
| To evaluate the role of platelet-rich plasma in healing of acute diaphyseal fractures of the femur. | /pubmed/28202370 | 1 | Chinese Journal of Traumatology | Musculoskeletal |
| Safety and efficacy of platelet-rich plasma in treatment of carpal tunnel syndrome; a randomized controlled trial. | /pubmed/29433485 | 1 | BMC Musculoskeletal Disorders | Musculoskeletal |
| Ultrasound guided injections of platelets rich plasma for muscle injury in professional athletes. Comparative study. | /pubmed/23702498 | 1 | Medical Ultrasonography | Musculoskeletal |
| Does platelet-rich plasma decrease time to return to sports in acute muscle tear? A randomized controlled trial. | /pubmed/27085364 | 1 | Knee Surgery, Sports Traumatology, Arthroscopy | Musculoskeletal |
| Relationship of cytokine levels and clinical effect on platelet-rich plasma-treated lateral epicondylitis. | /pubmed/28851099 | 1 | Journal of Orthopedic Research | Musculoskeletal |
| Platelet-rich plasma injections for the treatment of hamstring injuries: a randomized controlled trial. | /pubmed/25073598 | 2 | The American Journal of Sports Medicine | Musculoskeletal |
| Effect of single injection of platelet-rich plasma in comparison with corticosteroid on knee osteoarthritis: a double-blind randomized clinical trial. | /pubmed/26173792 | 1 | The Journal of Sports Medicine and Physical Fitness | Musculoskeletal |
| Platelet-rich plasma versus corticosteroid injection for recalcitrant lateral epicondylitis: clinical and ultrasonographic evaluation. | /pubmed/25920633 | 1 | Journal of Orthopaedic Surgery | Musculoskeletal |
| Comparison of autologous conditioned plasma injection, extracorporeal shockwave therapy, and conventional treatment for plantar fasciitis: a randomized trial. | /pubmed/23973504 | 1 | PM&R | Musculoskeletal |
| Treatment of knee osteoarthritis: platelet-derived growth factors vs. hyaluronic acid. A randomized controlled trial. | /pubmed/28783969 | 1 | Clinical Rehabilitation | Musculoskeletal |
| Intra-articular injection of photo-activated platelet-rich plasma in patients with knee osteoarthritis: a double-blind, randomized controlled pilot study. | /pubmed/26861957 | 1 | BMC Musculoskeletal Disorders | Musculoskeletal |
| Platelet-Rich Plasma Intra-articular Knee Injections Show No Superiority Versus Viscosupplementation: A Randomized Controlled Trial. | /pubmed/25952818 | 1 | The American Journal of Sports Medicine | Musculoskeletal |
| Clinical outcomes are associated with changes in ultrasonographic structural appearance after platelet-rich plasma treatment for knee osteoarthritis. | /pubmed/29878617 | 1 | International Journal of Rheumatic Diseases | Musculoskeletal |
| Hyaluronic Acid Versus Platelet-Rich Plasma: A Prospective, Double-Blind Randomized Controlled Trial Comparing Clinical Outcomes and Effects on Intra-articular Biology for the Treatment of Knee Osteoarthritis | /pubmed/28146403 | 1 | The American Journal of Sports Medicine. | Musculoskeletal |
| Platelet-rich plasma vs hyaluronic acid to treat knee degenerative pathology: study design and preliminary results of a randomized controlled trial. | /pubmed/23176112 | 2 | BMC Musculoskeletal disorder | Musculoskeletal |
| Platelet-rich plasma in patients with tibiofemoral cartilage degeneration. | /pubmed/23736793 | 1 | Archives of Orthopaedic and Trauma Surgery | Musculoskeletal |
| Platelet-rich plasma does not enhance return to play in hamstring injuries: a randomised controlled trial. | /pubmed/26136179 | 1 | British Journal of Sports medicine | Musculoskeletal |
| Is platelet-rich plasma able to enhance the results of arthroscopic microfracture in early osteoarthritis and cartilage lesion over 40 years of age? | /pubmed/23412171 | 1 | European Journal of Orthopaedic Surgery & Traumatology | Musculoskeletal |
| Platelet-Rich Plasma Augmentation of Arthroscopic Hip Surgery for Femoroacetabular Impingement: AÂ Prospective Study With 24-Month Follow-up. | /pubmed/25980923 | 2 | [Arthroscopy: The Journal of Arthroscopic & Related Surgery](https://www.sciencedirect.com/science/journal/07498063) | Musculoskeletal |
| Arthroscopic Debridement Versus Platelet-Rich Plasma Injection: A Prospective, Randomized, Comparative Study of Chronic Lateral Epicondylitis with a Nearly 2-Year Follow-Up. | /pubmed/28433443 | 2 | The Journal of Arthroscopy and Related Surgery | Musculoskeletal |
| Short-Term Outcomes of Percutaneous Trephination with a Platelet Rich Plasma Intrameniscal Injection for the Repair of Degenerative Meniscal Lesions. A Prospective, Randomized, Double-Blind, Parallel-Group, Placebo-Controlled Study. | /pubmed/30781461 | 1 | International Journal of Molecular Sciences | Musculoskeletal |
| Treatment of lateral epicondylitis with platelet-rich plasma, glucocorticoid, or saline: a randomized, double-blind, placebo-controlled trial. | /pubmed/23328738 | 1 | American Journal of Sports Medicine | Musculoskeletal |
| Rationale, secondary outcome scores and 1-year follow-up of a randomised trial of platelet-rich plasma injections in acute hamstring muscle injury: the Dutch Hamstring Injection Therapy study. | /pubmed/25940636 | 1 | British Journal of Sports medicine | Musculoskeletal |
| A Prospective, Randomized, Double-Blind, Parallel-Group, Placebo-Controlled Study Evaluating Meniscal Healing, Clinical Outcomes, and Safety in Patients Undergoing Meniscal Repair of Unstable, Complete Vertical Meniscal Tears (Bucket Handle) Augmented with Platelet-Rich Plasma. | /pubmed/29713647 | 1 | BioMed International Research | Musculoskeletal |
| No effects of PRP on ultrasonographic tendon structure and neovascularization in chronic midportion Achilles tendinopathy. | /pubmed/21047840 | 1 | British Journal of Sports medicine | Musculoskeletal |
| Plasma rich in growth factors (PRGF) as a treatment for high ankle sprain in elite athletes: a randomized control trial. | /pubmed/24938396 | 2 | Knee Surgery, Sports Traumatology, Arthroscopy | Musculoskeletal |
| Subacromial injection of autologous platelet-rich plasma versus corticosteroid for the treatment of symptomatic partial rotator cuff tears. | /pubmed/27544678 | 2 | European Journal of Orthopaedic Surgery & Traumatology | Musculoskeletal |
| Comparison of platelet-rich plasma and steroid injection in the treatment of plantar fasciitis. | /pubmed/25637732 | 2 | Acta Orthopaedica et Traumatologica Turcica | Musculoskeletal |
| The efficacy and safety of autologous conditioned serum (ACS) injections compared with betamethasone and placebo injections in the treatment of chronic shoulder joint pain due to supraspinatus tendinopathy: a prospective, randomized, double-blind, controlled study. | /pubmed/30167587 | 1 | Medical Ultrasonography | Musculoskeletal |
| Leukocyte-poor platelet-rich plasma versus bupivacaine for recalcitrant lateral epicondylar tendinopathy. | /pubmed/25920634 | 1 | Journal of Orthopaedic Surgery | Musculoskeletal |
| Intratendinous adipose-derived stromal vascular fraction (SVF) injection provides a safe, efficacious treatment for Achilles tendinopathy: results of a randomized controlled clinical trial at a 6-month follow-up. | /pubmed/28251260 | 1 | Knee Surgery, Sports Traumatology, Arthroscopy | Musculoskeletal |
| Magnetic resonance and ultrasound in Achilles tendinopathy: Predictive role and response assessment to platelet-rich plasma and adipose-derived stromal vascular fraction injection. | /pubmed/28987658 | 1 | European Journal of Radiology | Musculoskeletal |
| Platelet-Rich Plasma Injection with Arthroscopic Acromioplasty for Chronic Rotator Cuff Tendinopathy: A Randomized Controlled Trial. | /pubmed/26498958 | 1 | The American Journal of Sports medicine | Musculoskeletal |
| The effect of platelet-rich plasma on arthroscopic double-row rotator cuff repair: a clinical study with 12-month follow-up. | /pubmed/26969955 | 1 | Acta Orthopaedica et Traumatologica Turcica | Musculoskeletal |
| A Midterm Evaluation of Postoperative Platelet-Rich Plasma Injections on Arthroscopic Supraspinatus Repair: A Randomized Controlled Trial. | /pubmed/28806095 | 1 | American Journal of Sports Medicine | Musculoskeletal |
| Comparison of the therapeutic effects of ultrasound-guided platelet-rich plasma injection and dry needling in rotator cuff disease: a randomized controlled trial. | /pubmed/24938396 | 1 | Clinical Rehabilitation | Musculoskeletal |
| Platelet-rich plasma in rotator cuff repair: a prospective randomized study. | /pubmed/25086065 | 1 | The American Journal of Sports Medicine | Musculoskeletal |
| Greater Trochanteric Pain Syndrome: Percutaneous Tendon Fenestration Versus Platelet-Rich Plasma Injection for Treatment of Gluteal Tendinosis. | /pubmed/27663654 | 1 | Journal of Ultrasound in Medicine | Musculoskeletal |
| Sodium Hyaluronate and Platelet-Rich Plasma for Partial-Thickness Rotator Cuff Tears. | /pubmed/30199423 | 1 | Medicine and Science in Sports and Exercise | Musculoskeletal |
| A Pilot Study Evaluating the Effectiveness of Platelet-Rich Plasma Therapy for Treating Degenerative Tendinopathies: A Randomized Control Trial with Synchronous Observational Cohort. | /pubmed/26849812 | 1 | PLoS One | Musculoskeletal |
| One-year follow-up of platelet-rich plasma treatment in chronic Achilles tendinopathy: a double-blind randomized placebo-controlled trial. | /pubmed/21602565 | 1 | The American Journal of Sports Medicine | Musculoskeletal |
| Ultrasound-Guided Injection Therapy of Achilles Tendinopathy with Platelet-Rich Plasma or Saline: A Randomized, Blinded, Placebo-Controlled Trial. | /pubmed/27257167 | 1 | The American Journal of Sports medicine | Musculoskeletal |
| Platelet-rich plasma injections in the treatment of chronic rotator cuff tendinopathy: a randomized controlled trial with 1-year follow-up. | /pubmed/23893418 | 1 | The American Journal of Sports medicine | Musculoskeletal |
| Double-blind, Randomized, Placebo-controlled Study Evaluating the Use of Platelet-rich Plasma Therapy (PRP) for Acute Ankle Sprains in the Emergency Department. | /pubmed/26048069 | 1 | Journal of Emergency Medicine | Musculoskeletal |
| Effect of High-Volume Injection, Platelet-Rich Plasma, and Sham Treatment in Chronic Midportion Achilles Tendinopathy: A Randomized Double-Blinded Prospective Study. | /pubmed/28530451 | 1 | The American Journal of Sports Medicine. | Musculoskeletal |
| Comparison of Plantar Fasciitis Injected with Platelet-Rich Plasma vs Corticosteroids. | /pubmed/29600719 | 2 | Foot & Ankle International | Musculoskeletal |
| The effect of subacromial injections of autologous conditioned plasma versus cortisone for the treatment of symptomatic partial rotator cuff tears. | /pubmed/26017742 | 2 | Knee Surgery, Sports Traumatology, Arthroscopy | Musculoskeletal |
| Does Pure Platelet-Rich Plasma Affect Postoperative Clinical Outcomes After Arthroscopic Rotator Cuff Repair? A Randomized Controlled Trial. | /pubmed/27184542 | 1 | The American Journal of Sports medicine | Musculoskeletal |
| Magnetic resonance imaging evaluation of patellar tendon graft remodeling after anterior cruciate ligament reconstruction with or without platelet-rich plasma. | /pubmed/23629979 | 1 | Journal of Orthopedic Surgery | Musculoskeletal |
| Platelet-rich plasma injection in the treatment of frozen shoulder: A randomized controlled trial with 6-month follow-upâ€©. | /pubmed/29932415 | 1 | International Journal of Clinical Pharmacology and Therapeutics | Musculoskeletal |
| Platelet-rich plasma versus corticosteroid injections for carpal tunnel syndrome. | /pubmed/27921443 | 2 | Journal of Plastic Surgery and Hand Surgery | Neurology |
| Sensory improvement of leprosy peripheral neuropathy in patients treated with perineural injection of platelet-rich plasma. | /pubmed/24168291 | 1 | International Journal of Dermatology | Neurology |
| Six-month efficacy of platelet-rich plasma for carpal tunnel syndrome: A prospective randomized, single-blind controlled trial. | /pubmed/28273894 | 1 | Scientific Reports | Neurology |
| Effects of subtenon-injected autologous platelet-rich plasma on visual functions in eyes with retinitis pigmentosa: preliminary clinical results. | /pubmed/29546474 | 2 | Graefe's Archive for Clinical and Experimental Ophthalmology | Ophthalmology |
| Is arthrocentesis plus platelet-rich plasma superior to arthrocentesis plus hyaluronic acid for the treatment of temporomandibular joint osteoarthritis: a randomized clinical trial. | /pubmed/27364372 | 1 | International Journal of Oral and Maxillofacial Surgery | Oral Maxillofacial |
| Comparison of intra-articular injection of plasma rich in growth factors versus hyaluronic acid following arthroscopy in the treatment of temporomandibular dysfunction: A randomised prospective study. | /pubmed/28237253 | 1 | Journal of Cranio-Maxillofacial Surgery | Oral Maxillofacial |
| Platelet-Rich Plasma Injection as an Effective Treatment for Temporomandibular Joint Osteoarthritis. | /pubmed/25882438 | 1 | Journal of Oral and Maxillofacial Surgery | Oral Maxillofacial |
| Does injection of plasma rich in growth factors after temporomandibular joint arthroscopy improve outcomes in patients with Wilkes stage IV internal derangement? A randomized prospective clinical study. | /pubmed/26922496 | 1 | International Journal of Oral and Maxillofacial Surgery | Oral Maxillofacial |
| Intra-articular platelet-rich plasma injection for the treatment of temporomandibular disorders and a comparison with arthrocentesis. | /pubmed/25491276 | 1 | Journal of Cranio-Maxillofacial Surgery | Oral Maxillofacial |
| Is Arthrocentesis Plus Platelet-Rich Plasma Superior to Arthrocentesis Alone in the Treatment of Temporomandibular Joint Osteoarthritis? A Randomized Clinical Trial. | /pubmed/25976690 | 1 | Journal of Oral and Maxillofacial Surgery | Oral Maxillofacial |
| Application of platelet-rich plasma in plastic surgery: clinical and in vitro evaluation. | /pubmed/19231923 | 2 | Tissue Engineering Part C: Methods | Plastic Surgery |
| A comparative translational study: the combined use of enhanced stromal vascular fraction and platelet-rich plasma improves fat grafting maintenance in breast reconstruction. | /pubmed/23197813 | 2 | Stem Cells Translational Medicine | Plastic Surgery |
| The effect of coagulation protection with combination of epsilon aminocaproic acid and plasma saver in open-heart surgery. | /pubmed/9874863 | 1 | Acta anaesthesiologica Sinica | Cardiothoracic Surgery |
