# Supplementary material for: Reporting in clinical studies on platelet-rich plasma therapy among all medical specialties: A systematic review of Level I and II studies
Source: PLoS One. 2021 Apr 23;16(4):e0250007. doi: 10.1371/journal.pone.0250007 (PMC8064527; doi:10.1371/journal.pone.0250007)
Supplement: S2 Appendix — (DOCX) [file pone.0250007.s003.docx]

**Appendix 2.** Cochrane Risk of Bias Assessment

| Lead Author | Level Of Evidence | Randomization | Deviation from Intervention | Missing Outcome Data | Measurement of Outcome | Selection of Reported Result | Overall Bias |
| --- | --- | --- | --- | --- | --- | --- | --- |
| Simental-Mendía | 1 | Low | Low | Low | High | Low | High |
| Monto | 1 | Low | Low | Low | Some concerns | Low | Some concerns |
| Singla | 1 | Some concerns | Low | Low | Low | Some concerns | Some concerns |
| Acosta-Olivo | 1 | Low | Low | Low | Low | Low | Low |
| Gosens | 1 | Low | High | Low | Low | High | High |
| Wu | 1 | Low | Low | Low | Low | Low | Low |
| Kim | 1 | Some concerns | Low | Low | Low | Low | Some concerns |
| Mei-Dan | 1 | Some concerns | Low | Low | High | High | High |
| Dallari | 1 | Some concerns | Low | Low | Low | Low | Some concerns |
| Su | 1 | Low | Low | Low | High | High | High |
| Cerza | 1 | Some concerns | Low | Low | Low | Some concerns | Some concerns |
| Duymus | 1 | Low | High | High | High | High | High |
| Sante | 1 | Low | Low | Low | Some concerns | Low | Some concerns |
| Montañez-Heredia | 1 | Low | Low | Low | Low | High | High |
| Papalia | 1 | Low | High | Low | Low | High | High |
| Mahindra | 1 | Low | Low | High | Low | High | High |
| Battaglia | 1 | Some concerns | Low | Low | Some concerns | High | High |
| Lebiedziński | 1 | Some concerns | High | High | Some concerns | High | High |
| Tuakli-Wosornu | 1 | Low | Some concerns | Some concerns | Low | High | High |
| Smith | 1 | Low | Low | Low | Low | Low | Low |
| Wu | 1 | Low | Low | Low | Low | High | High |
| Görmeli | 1 | Low | Low | Some concerns | Low | High | High |
| Patel | 1 | Low | Low | Low | Low | High | High |
| Montalvan | 1 | Some concerns | Low | Some concerns | Low | Low | Some concerns |
| Görmeli | 1 | Some concerns | High | High | Low | High | High |
| Thanasas | 1 | Low | Some concerns | Low | Low | High | High |
| Vetrano | 1 | Low | Low | Low | Some concerns | High | High |
| Faghihi | 1 | Low | Low | Some concerns | Some concerns | Low | Some concerns |
| Dragoo | 1 | Low | Low | Low | Low | Low | Low |
| de Vos | 1 | Low | Low | Low | Low | Low | Low |
| Creaney | 1 | Low | High | High | Low | Low | High |
| Davenport | 1 | Some concerns | Low | Some concerns | Low | Low | Some concerns |
| Samuel | 1 | High | Low | Low | Low | Low | High |
| Singh | 1 | Low | Low | Low | Low | Low | Low |
| Trink | 1 | Low | Low | High | Low | High | High |
| Gentile | 1 | Low | Low | Low | Low | High | High |
| Mapar | 1 | Low | Low | Low | Low | Low | Low |
| Raeissadat | 1 | Low | Low | Low | Some concerns | Low | Some concerns |
| Bubnov | 1 | Some concerns | Low | Low | High | High | High |
| Jain | 1 | Some concerns | High | High | High | High | High |
| Rossi | 1 | Low | Low | Low | Some concerns | Low | Some concerns |
| Lim | 1 | Low | High | High | Some concerns | High | High |
| Forogh | 1 | Low | Low | High | Low | High | High |
| Duif | 2 | Low | Low | Some concerns | Low | Low | Some concerns |
| Schöffl | 2 | Low | Low | High | Low | Low | High |
| Hamid | 2 | Low | High | High | Low | Low | High |
| Filardo | 2 | Low | High | Low | Low | Low | High |
| Rafols | 2 | Low | Low | High | Low | High | High |
| Laver | 1 | Low | Low | Low | Low | Low | Low |
| Shams | 2 | Low | Some concerns | Low | Low | High | High |
| Jain | 2 | Low | Low | Low | Some concerns | Low | Some concerns |
| Zhang | 1 | Low | Low | Low | Low | High | Some concerns |
| Fontdevila | 2 | Low | Low | Some concerns | Low | Low | Some concerns |
| Morella | 2 | Low | Low | Low | Some concerns | Low | Some concerns |
| Uğurlar | 1 | Low | Low | Low | Low | Low | Low |
| Rodrigues | 1 | Some concerns | Low | Low | Low | High | Some concerns |
| Liu | 1 | Low | Low | Low | Low | Low | Low |
| Shetty | 2 | Low | Low | Low | Low | High | High |
| Gautam | 1 | Low | High | Low | Low | Low | High |
| Chew | 1 | Low | Low | Low | Low | Low | Low |
| Lisi | 1 | Low | Low | Low | Low | Low | Low |
| Paterson | 1 | Some concerns | Low | Low | Low | Low | Some concerns |
| Filardo | 1 | Some concerns | Some concerns | Low | Low | Low | Some concerns |
| Ahmad | 1 | Low | High | High | Low | Low | High |
| Cole | 1 | Low | Some concerns | Low | Low | Low | Some concerns |
| Hart | 1 | Low | Low | Low | Low | Low | Low |
| Hamilton | 1 | Low | Low | Low | Low | Low | Low |
| Anjayani | 1 | Low | High | Low | High | Low | Low |
| Hancı | 1 | Low | Low | Low | Low | Low | Low |
| Kilic | 1 | Low | Low | Low | High | Low | Some concerns |
| Lee | 1 | Some concerns | Low | Low | High | Low | High |
| Abdelghani | 1 | Some concerns | High | Low | Low | Low | High |
| Kaminski | 1 | Low | Low | Low | Low | Low | Low |
| Kilic | 1 | Low | High | Low | Low | Low | High |
| Krogh | 1 | Low | High | Low | High | Low | High |
| Reurink | 1 | Low | Low | Low | Low | Low | Low |
| Kaminski | 1 | Low | High | High | Low | Low | Some concerns |
| Lee | 1 | High | Low | High | Low | Low | High |
| Fernández Sanromán | 1 | Low | Low | Low | Low | Low | Low |
| Wu | 1 | Low | Low | Low | Low | Low | Low |
| de Vos | 1 | Low | Low | Low | Low | Low | Low |
| Damjanov | 1 | Some concerns | Low | Low | High | Low | High |
| Fernández-Ferro | 1 | Low | Low | Low | Some concerns | Low | Some concerns |
| Hegab | 1 | Low | Some concerns | Low | Low | Low | Some concerns |
| Behera | 1 | Low | Low | Low | Low | Low | Low |
| Usuelli | 1 | Low | Low | Low | Low | Low | Low |
| Albano | 1 | Low | Low | Low | Low | Low | Low |
| Carr | 1 | Low | Low | Low | Low | Low | Low |
| Ebert | 1 | Low | Low | Low | High | Low | High |
| Laver | 2 | Some concerns | Low | Low | High | Low | High |
| Malavolta | 1 | Low | Low | Low | Low | Low | Low |
| Jacobson | 1 | Low | Low | Low | High | Low | High |
| Cai | 1 | Low | High | High | Low | Low | High |
| Wesner | 1 | Some concerns | High | Low | Low | Low | High |
| de Jonge | 1 | Low | Low | Low | Low | Low | Low |
| Krogh | 1 | Low | High | High | High | Low | High |
| Kesikburun | 1 | Low | Low | Low | Low | Low | Low |
| Rowden | 1 | Low | High | Low | Low | Low | High |
| Boesen | 1 | Low | Low | Low | Low | Low | Low |
| Flury | 1 | Low | High | Low | Low | Low | Low |
| Seijas | 1 | Low | Low | Low | Low | Low | Low |
| Tawfik | 1 | Low | Low | Low | Low | Low | Low |
| Puig | 1 | Low | High | Low | Low | Low | High |
| Taieb | 1 | Low | High | Low | High | Low | High |
| Ibrahim | 1 | Low | Low | Low | High | Low | Some concerns |
| Lin | 1 | Low | Low | Low | Some concerns | Low | Some concerns |
