# Supplementary material for: Reporting in clinical studies on platelet-rich plasma therapy among all medical specialties: A systematic review of Level I and II studies
Source: PLoS One. 2021 Apr 23;16(4):e0250007. doi: 10.1371/journal.pone.0250007 (PMC8064527; doi:10.1371/journal.pone.0250007)
Supplement: S3 Appendix — (DOCX) [file pone.0250007.s004.docx]

| **Lead author name** | **Level of Evidence** | **MINORS Score** |
| --- | --- | --- |
| Schiavone | 2 | 20 |
| Abdel aal | 2 | 11 |
| Nofal | 2 | 11 |
| Gawdat | 2 | 21 |
| Nofal | 2 | 19 |
| Ibrahim | 2 | 16 |
| Hodeib | 2 | 18 |
| Gamil | 2 | 20 |
| Ibrahim | 2 | 20 |
| Chakravdhanula | 2 | 20 |
| Redmond | 2 | 15* |
| Mishra | 2 | 18 |
| Filardo | 2 | 19 |
| Kon | 2 | 15 |
| Ertugrul | 2 | 20 |
| Spakova | 2 | 17 |
| Say | 2 | 15 |
| Tetschke | 2 | 16 |
| Say | 2 | 19 |
| von Wehren | 2 | 21 |
| Rahimzadeh | 2 | 17 |
| Bastos | 2 | 19 |
| Uzun | 2 | 17 |
| Arslan | 2 | 18 |
| Cervelli | 2 | 11 |
| Gentile | 2 | 18 |

**Appendix 3.** Critical Appraisal of Nonrandomized Studies

MINORS (Methodological Index for Nonrandomized Studies) score for comparative studies out of 24.*Criteria 6, 7 did not apply.
